# Assessing the magnitude of changes from protocol to publication—a survey on Cochrane and non-Cochrane Systematic Reviews

**DOI:** 10.7717/peerj.16016

**Published:** 2023-10-02

**Authors:** Maximilian Siebert, Laura Caquelin, Meisser Madera, Roberto Acosta-Dighero, Florian Naudet, Marta Roqué

**Affiliations:** 1 Stanford University, Meta-Research Innovation Center at Stanford (METRICS), Stanford University, Stanford, CA, USA; 2 Université de Rennes, Univ Rennes, CHU Rennes, Inserm, Centre d’investigation clinique de Rennes (CIC1414), Service de pharmacologie clinique, Institut de recherche en santé, environnement et travail (Irset), UMR S 1085, EHESP, Rennes, France; 3 Faculty of Dentistry at the University of Cartagena, Department of Research, Cartagena, Colombia; 4 Universidad de Valparaíso, Interdisciplinary Centre for Health Studies (CIESAL), Valparaíso, Chile; 5 Institut Universitaire de France, Paris, France; 6 Sant Pau Biomedical Research Institute (IIB-Sant Pau), Iberoamerican Cochrane Centre, Barcelona, Spain; 7 CIBER of Epidemiology and Public Health (CIBERESP), Madrid, Spain

**Keywords:** Systematic reviews, Pre-registration, Protocols, Cochrane, Reporting bias

## Abstract

**Objective:**

To explore differences between published reviews and their respective protocols in a sample of 97 non-Cochrane Systematic Reviews (non-CSRs) and 97 Cochrane Systematic Reviews (CSRs) in terms of PICOS (Patients/Population, Intervention, Comparison/Control, Outcome, Study type) elements and the extent to which they were reported.

**Study Design and Setting:**

We searched PubMed and Cochrane databases to identify non-CSRs and CSRs that were published in 2018. We then searched for their corresponding Cochrane or PROSPERO protocols. The published reviews were compared to their protocols. The primary outcome was changes from protocol to review in terms of PICOS elements.

**Results:**

We identified a total of 227 changes from protocol to review in PICOS elements, 1.11 (Standard Deviation (SD), 1.22) changes per review for CSRs and 1.23 (SD, 1.12) for non-CSRs per review. More than half of each sub-sample (54.6% of CSRs and 67.0% of non-CSRs) (Absolute Risk Reduction (ARR) 12.4% [−1.3%; 26.0%]) had changes in PICOS elements. For both subsamples, approximately a third of all changes corresponded to changes related to primary outcomes. Marked differences were found between the sub-samples for the reporting of changes. 95.8% of the changes in PICOS items were not reported in the non-CSRs compared to 42.6% in the CSRs (ARR 53.2% [43.2%; 63.2%]).

**Conclusion:**

CSRs showed better results than non-CSRs in terms of the reporting of changes. Reporting of changes from protocol needs to be promoted and requires general improvement. The limitations of this study lie in its observational design. Registration: https://osf.io/6j8gd/.

## Introduction

Systematic reviews (SRs) play an essential role inside and outside biomedical research. Because of their high standing in the pyramid of evidence, their results can change how medical research is conducted, how clinical decisions are reached, how policies are designed, and how patients are informed about the benefits and risks of therapeutic interventions (Mulrow, 1994; Murad et al., 2016; Cook, Mulrow & Haynes, 1997).

SRs in the Cochrane framework are pivotal in this process. This is linked to the development of helpful guidelines and tools for the conduct of SRs, such as bias assessments, and the publication of the Cochrane Handbook (Higgins et al., 2023). Cochrane Systematic Reviews (CSRs) are often described as the gold standard of SRs and enjoy a high level of confidence (Useem et al., 2015; Rosenbaum, Glenton & Cracknell, 2008).

The pre-registration of protocols is considered as methodologically important and especially so because it is considered as an instrument to avoid reporting bias (Richards & Onakpoya, 2019). Furthermore, it increases transparency in the details provided on the research and on subsequent changes from the methods planned. Thus peer-reviewers are able to consult the protocol and get a better understanding of the research project. Today, pre-registrations have reached such standing that several journals, such as the PLoS journals or the BMJ, include the registration details in their instructions to future authors, who then incorporate them in the final manuscripts (Stewart, Moher & Shekelle, 2012).

In addition to the Cochrane Database of Systematic Reviews (CDSR), SRs focusing on health outcomes can be prospectively registered on PROSPERO, which was launched in 2011 as a free, open, online tool, where researchers can register their protocols in a database before starting the actual SR (University of York, 2023). Besides the initiative to promote open research, this platform has the advantage of assigning each protocol to a unique identifier to facilitate its identification and transparency.

Following the above-mentioned initiatives, numerous studies have been able to assess discrepancies between the original protocols and the published SRs in the biomedical field—for both CSRs and non-Cochrane SRs (non-CSRs) (Silagy, Middleton & Hopewell, 2002; Tricco et al., 2016; Pandis et al., 2015).

However, most studies that have looked at the differences between the registered protocols and the published SRs have focused on changes in primary outcomes (Tricco et al., 2016; Dwan et al., 2013). While acknowledging the paramount importance of these characteristics in an SR, it is crucial to emphasize the significance of all elements that constitute the PICOS method (Patients/Population, Intervention, Comparison/Control, Outcome, and Study type).

Each component plays a vital role in ensuring a comprehensive and rigorous analysis.

In an article by Tricco et al. (2016) examining published reviews and their protocol registered on PROSOERO, they found that a high percentage of reviews did not explicitly specify a primary outcome.

Despite these discrepancies, there was no statistically significant association found between discrepant outcome reporting and having a favorable and statistically significant meta-analysis result or positive conclusion. However, as the authors state, the limited number of reviews within each subgroup of discrepancy classification may have impacted the statistical power to detect significant results. Aware of these issues, Cochrane updated its Handbook. Since 2008, a requirement to report changes from protocol to review have been included (Higgins et al., 2023).

Since 2013, the Cochrane Editorial Unit has conducted external screening to ensure that any changes made to systematic reviews are documented and justified in new reviews published after September 2013 (Pandis et al., 2015). This has helped to maintain transparency and accountability in the review process.

The aim of this study was to investigate whether the new measures have brought any improvement in the quality of CSRs by reducing the discrepancies between CSR protocols and SRs, as compared to SRs published elsewhere and not constrained by such requirements. To this end, we assessed the frequency and reporting of changes in PICOS elements from protocol to SR and the reporting of these changes, in a sample of SRs published in 2018, and compared the results to non-CSRs registered on PROSPERO.

## Methods

### Study design and registration

The design of this study was a survey of SRs, comparing CSRs and non-CSRs published in 2018 to their pre-registered protocols, with a focus on the PICOS method. The aims and the methods of this study were described prior to the study in a protocol registered on 25/09/2019 on the Open Science Framework: https://osf.io/6j8gd/.

### Inclusion criteria

We included SRs with or without meta-analyses assessing any therapeutic intervention in any disease, widely known as intervention SRs (Higgins et al., 2023), if they had a registered protocol on either CDSR or PROSPERO, and were published for the first time between 01/01/2018 and 01/01/2019. Our goal was to obtain a current snapshot of the Systematic Review landscape.

We excluded other types of SRs, like diagnostic test accuracy reviews or prognosis reviews. We did so in order to include reviews that assess the effectiveness/safety of a treatment, vaccine, device, preventative measure, procedure or policy and are the most relevant to the field of the authors. For each SR selected, the protocol was retrieved. SRs and their protocols were to be published in English to facilitate extraction across the international team.

When several versions of the same SR were identified, we only considered the most recent.

### Electronic searches

We searched for CSRs in the CDSR (via the Cochrane Library). This database contains all CSRs and features a tool that enables searches according to review type and publication date. We used these filters to identify potential intervention CSRs to be included in our study. This search was conducted on 02/09/2019. Furthermore, only CSRs with a protocol were eligible.

Likewise, we searched non-CSRs (PROSPERO SRs) on Medline (via PubMed). In order to identify non-CSRs on the intervention type from this database, we used a search strategy designed by an expert in this field (Table S1). In brief, it enables the identification of SRs on PubMed that have a PROSPERO CDR registration number. This number then enables the retrieval of the original protocol on PROSPERO. This search was conducted on 10/09/2019.

### Selection of reviews

For the CSRs, we imported all retrieved records on an Excel sheet and sorted them in a random order. Similarly, for the non-CSRs, one author (M.S.) screened the results of the search by title and abstract, to exclude irrelevant studies. Full-text SRs were obtained for further assessment to determine final inclusion according to the eligibility criteria. A random selection of the sample was performed with the rnorm() function in R (R Core Team, 2021).

### Hypotheses & sample size calculation

By conducting a literature search, we found that 30.1% of CSRs showed differences in outcomes (Pandis et al., 2015) from the protocol to the SR, while around 62.5% of non-CSRs showed differences in eligibility criteria (Koensgen et al., 2019). After consulting with the team, we decided to account for uncertainties, possible errors and possible overestimation of such an estimated difference of 31.5%. We therefore decided to power the study in order to be able to detect a smaller difference of 20% in a two-tailed test with alpha = 0.05 and beta = 0.20.

Our assumptions were based on proportions of 60% and 40%, as previously explained.

Utilizing the chi-square test, we calculated that a total of 194 SRs (97 CSRs and 97 non-CSRs) would be necessary to achieve the required power to test our hypotheses.

### Quality assessment of the SRs included

To assess the methodological quality of the SRs, we used the AMSTAR-2 (A Measurement Tool to Assess Systematic Reviews-2). It is composed of 16 items, among which seven are critical domains. There are four levels to be distinguished: high, moderate, low and critically low (Shea et al., 2017).

For a SR to get a high score, no or one non-critical weakness should be found, meaning that the SR provided “an accurate and comprehensive summary of the results of the available studies that address the question of interest”.

For moderate quality, more than one non-critical weakness and for low one critical flaw with or without non-critical weaknesses must be found.

Regarding a score of critically low quality, more than one critical flaw with or without non-critical weaknesses needs to be found. The latter is by the authors described as an SR that “has more than one critical flaw and should not be relied on to provide an accurate and comprehensive summary of the available studies”.

The quality of SRs was assessed by two reviewers (M.S. or M.R.) in a single extraction.

### Data collection

Before data collection, all reviewers (M.S., L.C., R.D. or M.M.) completed a pilot sample of three studies for each review type. At least two of the reviewers extracted data independently, and any discrepancies were resolved by consensus. In case of disagreement, a third reviewer arbitrated (M.R.).

We extracted the following general characteristics of the SRs: changes in protocol title and SR title, changes in first author between protocol and SR, country of affiliation of first author, country of affiliation of corresponding author, publication date of protocol, dates of changes/searches (if indicated), publication date of SR.

Furthermore, any changes from protocol to SR on PICOS items were collected. Finally changes in secondary outcomes and reporting of the changes from protocol to SR were explored.

Declaration of changes could be found on the information page along with the published website for CSRs. For non-CSRs, we looked if this was stated on the respective PROSPERO site or in the publication.

Our primary outcome was change from protocol to review in terms of PICOS elements. Our secondary outcomes were changes from protocol to SR in the secondary outcomes, the reporting in the published SRs of changes, the time between protocol and SR publication, the time between the last searches and SR publication, and SR quality.

For any given review, all changes in PICOS characteristics were assessed in terms of the magnitude of change via an *ad-hoc* exploratory scale that classified changes into small, moderate, or major in terms of their relevance within the SR (see Table 1). By applying this scale, we gave priority to crucial changes, such as changes in the population reported in the final article, over the reporting of less important changes from protocol. The classification was conducted in agreement between researchers on the perceived relevance of the change in relation to the review.

### Data management and synthesis

For the extracted variables, we calculated the means and their corresponding standard deviations. We utilized the Chi-square test to compare the outcomes’ proportions for the PICOS changes.

Estimates of association were computed between the time in months from the protocol to the publication and changes in the primary outcome measures from the protocol to the study report, with regard to the study population, comparisons, and outcomes.

The shortfall between non-CSRs and CSRs in terms of changes and their reporting was assessed with the absolute risk reduction (ARR) method and the respective 95% Confidence Interval. For details on the calculation of the ARR and its 95% confidence intervals, please refer to the Supplementary Material.

In order to determine the presence of a statistically significant difference between the CSR and non-CSR sub-samples, corresponding *p*-values were calculated. However, this analysis was exclusively conducted for the PICOS characteristics.

The analyses were conducted in R Version 4.3.0 (R Core Team, 2021), R Studio Version 1.4.1106 (RStudio Team, 2020). The used non-base packages of the free software program were: readxl Version 1.4.2 (Wickham & Bryan, 2023), ggplot2 Version 3.4.2 (Wickham et al., 2023a), dplyr Version 1.1.2 (Wickham et al., 2023b), plyr Version 1.8.8 (Wickham, 2022), tidyverse Version 2.0.0 (Wickham, 2023), zoo Version 1.8-12 (Zeileis et al., 2023), prettyR Version 2.2-3 (Lemon & Grosjean, 2019) and Rmisc Version 1.5.1 (Hope, 2022) and can also be found in the code that we uploaded to the OSF website.

### Deviations from protocol to SR publication

The deviations from protocol to publication can be found in the Supplementary Material.

## Results

### Search results and description of the sample

**Figure 1:**
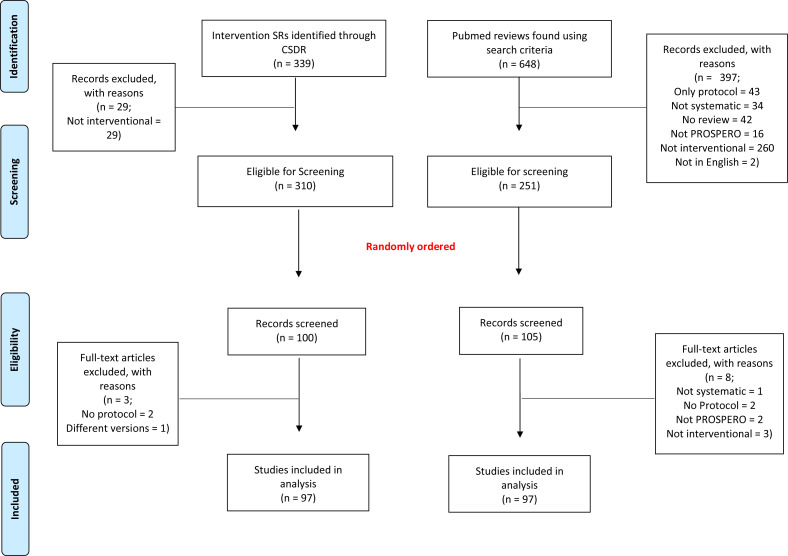
Flowchart of our selection process for systematic reviews.

The selection process is reported in Fig. 1. Briefly, for CSRs, we identified 339 records. Of these, 310 (91.4%) were considered as intervention SRs. A total of 97 SRs were randomly selected and included in our analyses. Likewise, for non-CSRs, our systematic literature search retrieved 648 records. Of these, 397 were excluded, mainly for not being interventional SRs, resulting in a total of 251 (38.7%) eligible SRs, from which the final sample of 97 non-CSRs was randomly selected.

The list of reviews, excluded and included, reviews can be found on the Open Science Framework website (https://osf.io/5ywus/).

Out of the 194 SRs included, more than 70% in each sample had a first author from a high-income region: 76/97 (78.4%) among CSRs and 69/97 (71.1%) among non-CSRs. The same was true for the corresponding authors (78.4% and 72.2%, respectively).

Time between the publication of the protocol to the final published review was 31.4 (SD, 24.7) months on average. The time to publish for CSRs was longer, with an average duration of 41.1 months (SD, 29.5), compared to non-CSRs, which took an average of 21.8 months (SD, 12.7). Conversely, the time between the date of publication and the last amendments to the protocol was shorter for CSRs than for non-CSRs: 1.4 (SD, 2.4) months vs 13.1 (SD, 10.7) months.

### Comparison of protocols with their published systematic reviews

Overall, our research yielded that out of 97 CSRs, 32 (33.0%) had no change between protocol and SR, 53 (54.6%) that included a change in PICOS elements, and 12 (12.3%) that included other changes. For the 53 CSRs that involved changes in PICOS elements, more than half (28 SRs, 52.8%) had at least one major change. Out of these 28 SRs, 10 (35.7%) reported all changes (major, moderate and small), and 19 (67.9%) reported all major changes.

Among the 97 non-CSRs, we found 24 SRs (24.7%) that had no changes from protocol, 65 (67.0%) that had changes in PICOS elements, and 8 SRs with no changes in PICOS outcomes. Concerning the 65 remaining SRs, 39 SRs (60.0%) had at least one major change; of these 39 SRs, none reported all changes and only two (5.1%) reported all major changes.

**Figure 2:**
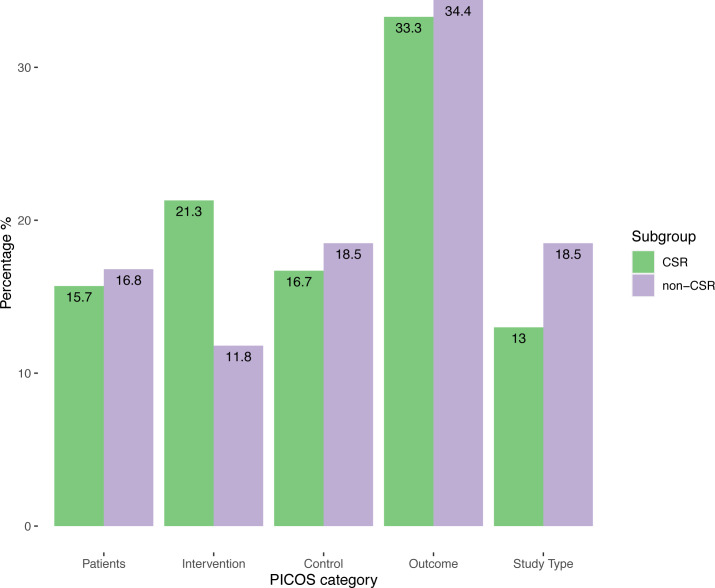
Distribution of changes according to PICOS elements and review type.

Quantification of the changes in PICOS elements made it possible to identify a total of 227 changes, 108 (47.6%) in the CSRs and 119 (52.4%) in the non-CSRs. 1.11 (SD, 1.22) changes per review for CSRs and 1.23 (SD, 1.12) for non-CSRs per review were registered. Following breakdown of the distribution of the changes according to the PICOS elements, we observed that for both samples approximately a third of the total changes corresponded to changes related to primary outcomes. These results can be found in Fig. 2.

With respect to the magnitude of change, in CSRs the largest proportion of the 108 changes in the CSRs 46/108 were small (42.6%), and major changes, 35/108 (32.4%), made up approximately a third of the total. Non-CSRs had the largest share of changes in the major change section 46/119 (38.7%).

**Table 1:** Examples of the classification of changes.

**Magnitude of Change**	**Examples**
Small	– changing the definition of an outcome/intervention, but only to make it clearer or more complete
Moderate	– adding or deleting a secondary outcome – changing study type inclusion criteria – reporting only on severe adverse effects, although separate reporting for general adverse effects and severe adverse effects was planned
Major	– adding or eliminating a primary outcome – setting up composite endpoints instead of individual ones – changing a comparator intervention – changing the population of interest in the SR

When looking at the reporting of all changes, in 46/108 (42.6%) of CSRs and in 114/119 (95.8%) of non-CSRs changes were not reported (ARR 53.2% [43.2%; 63.2%]). For both samples, the lowest reporting rate was found for small changes.

Details of the percentages, ARR-values and *p*-values for changes in relation to the magnitude of changes and the reporting of changes are shown in Table 2.

**Table 2:** Distribution of changes in PICOS elements according to magnitude of change, reporting status and review type.

Total changes in PICOS	CSRs (*n* = 108)	Non-CSRs (*n* = 119)	ARR	95% CI	*p*-value (Chi-square)
	Count (%)				
Small changes	46 (42.6%)	34 (28.6%)	−14.0%	[−26.4%; −1.7%]	0.04
Not Reported	24 (52.2%)	34 (100%)	47.8%	[33.4%; 62.3%]	*p* < 0.001
Moderate changes	27 (25.0%)	39 (32.7%)	7.7%	[−4.0%; 19.5%]	0.25
Not Reported	12 (44.4%)	36 (92.3%)	47.9%	[27.3%; 68.4%]	0.004
Major changes	35 (32.4%)	46 (38.7%)	6.3%	[−6.2%; 18.7%]	0.40
Not Reported	10 (28.6%)	44 (95.7%)	67.1%	[51.0%; 83.2%]	*p* < 0.001

**Notes.**

PICOS Patients, Intervention, Comparison/Control, Outcome, Study type CSR Cochrane Systematic Review ARR Absolute Risk Reduction CI Confidence Intervals)

Regarding changes not related to PICOS elements, out of the 97 CSRs included, 21/97 (21.6%) had a change in first author, compared to non-CSRs with 22/97 (22.7%); (ARR 1.1% [−10.7%; 12.7%]).

For changes in the SR title, 30/97 (30.9%) of CSRs and 54/97 (55.7%) of non-CSRs (ARR 24.8% [11.2%;38.2%]) had a change.

Regarding the secondary endpoints, we identified one or more changes in 46 of the 97 CSRs (47.4%) and in 43 of the 97 non-CSRs (44.3%) (ARR 3.1% [−9.8%; 18.0%]). Moderate changes were observed more often in non-CSRs than in CSRs (93.0% vs. 80.4%) (ARR 12.6% [−1.2%;26.4%]) and small changes appeared less often because we only distinguished these two types of changes.

Non-reporting of changes in the secondary outcomes, without distinction for small, moderate or major changes, was considerably more frequent among the non-CSRs 42/43, with only one non-CSR reporting any compared to less than half the CSRs non-reporting changes 20/46 (97.7% vs. 43.5% (ARR 54.2% [53.7%; 54.7%]).

### Methodological quality of the reviews in relation to changes

For the methodological quality of the SRs, 100% of CSRs achieved the highest ranking, while the quality of the non-CSRs was heterogeneous, with over half 49/97 (50.5%) classified as low quality and 21/97 (21.6%) as critically low.

**Figure 3:**
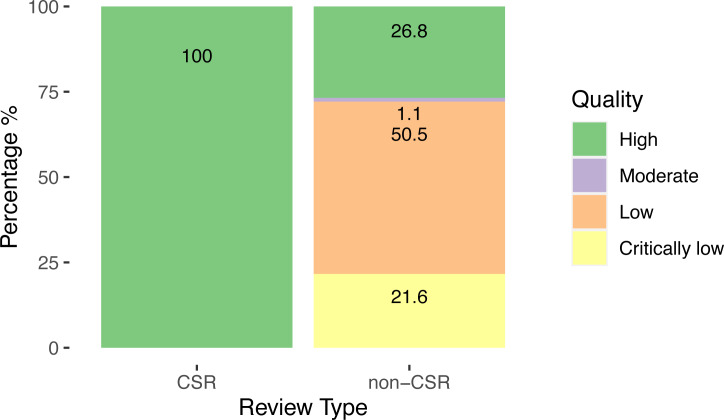
Quality of systematic reviews assessed by AMSTAR-2.

Our results are shown in Fig. 3.

There was a clear association between lower methodological quality of non-CSRs and the occurrence of changes from protocol, with lower-quality reviews being more likely to contain changes than higher-quality reviews. The same pattern was found across the four possible magnitudes of change (Fig. S1).

Methodological quality was also related to the reporting of changes from protocol. Non-CSRs with non-reported changes were mostly of low quality (Fig. S2).

## Discussion

### Summary of main findings

In this study we compared 97 non-CSRs and 97 CSRs that focused on any therapeutic interventions and were published in 2018. Changes between the protocols and the published SRs were described in terms of PICOS elements, non-PICOS elements and secondary outcomes.

More than half of each sub-sample, 54.6% of CSRs and 67.0% of non-CSRs had changes in PICOS elements (ARR 12.4% [−1.3%; 26.0%]). Furthermore, we found that most changes related to the primary outcome whatever the type of SR.

Overall, we found similar proportions for major changes in CSRs and non-CSRs (32.4% vs 38.7%) (ARR 6.3% [−6.2%;18.7%]). However, manifest differences were found between the sub-samples with regard to the reporting of changes. Only 3/119 (4.2%) of changes in PICOS items were reported in non-CSRs, compared to 62/108 (57.4%) in CSRs (ARR 53.2% [43.2%; 63.2%]).

A similar pattern was observed with regard to changes reported for the secondary outcomes.

Finally, we observed a significant difference in the occurrence of small changes, with CSRs containing more numerous changes than non-CSRs (42.6% vs. 28.6%) (ARR −14.0% [−26.4%; −1.7%]).

These finding suggest that there are still several deviations from registered protocols that are not described or justified in published SRs. Applying the AMSTAR-2 tool, we observed 100% high quality classification for CSRs, whereas more than two-thirds (72.1%) of non-CSRs were classified low or even critically low quality.

### Comparison of our findings with other studies

Several studies have focused only on certain outcomes in either non-CSRs or CSRs (Kirkham, Altman & Williamson, 2010; Parmelli, Liberati & D’Amico, 2007; Page et al., 2014) and studies comparing the two sub-groups are rare. In addition, we adopted a new type of approach and created our own Likert scale to assess the magnitude of changes. Therefore, it is somewhat difficult to compare our findings to other studies. Nevertheless, we have endeavored to put our research in context by comparing partial results to other papers.

In a study in 2014, Page and colleagues conducted a meta-analysis of four studies including 485 CSRs and found that 38% of the studies made a change to at least one primary outcome (Page et al., 2014). Our numbers are similar to these findings in 2014, although Page’s results were only valid for CSRs, and the study was conducted before the requirements of reporting of changes by Cochrane was mandatory.

Koensgen et al. (2019) checked for several PRISMA items when comparing protocol and final SR, and they did not focus solely on primary outcomes. However, they explored only non-CSR reviews. A total of 92.5% of the reviews involved a change, almost 50% included a major change for an item in the PRISMA list and only 10% of the changes were reported. The percentage of changes reported in non- CSRs in this study was higher than in ours (10% vs. 4.2%). One difference from our approach was the distinction between major and minor changes only, whereas we introduced a third category of moderate changes. Consequently, we present a lower rate of major changes in non-CSRs. While the definitions vary, the findings show that there is a real issue with the reporting of major changes in non-CSRs.

In a recent study by Hu et al. (2021), which focused on changes in PICOS outcomes from protocol to review in PROSPERO-registered reviews, the researchers found that 90% of the reviews underwent one change and 59% at least two changes. In line with our results, they found very low rates for reporting of changes and observed that the main area in SRs prone to changes concerned the outcomes (Hu et al., 2021).

The conclusions of the above-mentioned studies confirm our impression that changes are insufficiently reported and that there is a huge difference for SRs produced outside the Cochrane framework. Furthermore, they show that the numbers of changes in primary outcomes have remained at the same level over recent years. Creating awareness of the lack of reporting on deviations from protocol should thus be a priority.

### Strengths and limitations

Among the strengths of this study, we would like to stress that this was a first approach to the research question, in the form of a cross-sectional study including a representative sample of non-CSRs and CSRs. Furthermore, a sensitive search strategy was performed to identify non-CSRs. In addition, more than one reviewer independently conducted the whole selection process and data extraction from the SRs included. We also included an objective, widely used tool for assessing the quality of the SRs. We did not only focus on primary and secondary outcomes only, but we tried to have a more inclusive overview by observing PICOS characteristics. Finally, all our methods were specified a priori in a protocol.

Nonetheless, certain limitations to this study should be considered. Firstly, due to lack of resources, we were not able to contact all the authors and query them about our findings which would have cleared some issues. Secondly, detecting changes was easier when they were reported, as was the case for CSRs.

Indeed, the data extractors (mainly early career researchers) may have judged changes too strictly in some cases, and they may not have been experienced enough to know how difficult it is to be transparent in research. This also applies to the AMSTAR-2 assessment of SR quality. We only included SRs in English. Had other languages been included, our analysis might have looked different.

One missed opportunity in our study was the assessment of publication bias. Specifically, we could have examined whether subsequent publications would have followed if we had initiated our research with the respective protocols. This analysis would have provided valuable insights into the potential influence of publication bias on our findings.

Finally, our identification of changes in PICOS factors between protocols and SRs in an observational study setting is only exploratory. Numerous unmeasured confounders, for example the journals’ word count restrictions, unclear author instructions or changes during peer-review, could account for some of the associations found. There may be other confounders, and caution is warranted in interpreting these results.

### Implications for practice

We would encourage journals and publishers to generate recommendations that provide for differences from protocol to review to be indicated. Should the patterns observed in our investigation persist, reporting biases and unrealistic research standards will result. A possible solution to the issue could be that journals assign one peer-reviewer to the task of checking the differences in relation to the protocols (TARG Meta-Research Group Collaborators, 2022). Given the advancements in artificial intelligence, an alternative approach could be to utilize specialized software capable of detecting unreported changes automatically. For instance, large language models like ChatGPT have demonstrated their capabilities in performing systematic reviews, although they are still undergoing refinement and improvement in their beta phase (Qureshi et al., 2023). Detecting changes from a registered protocol would be the logical next step.

## Conclusions

The reporting of differences between protocol and publication in CSRs and non-CSRs has not improved compared to earlier studies. Despite new rules in the Cochrane framework and initiatives such as PROSPERO, there is room for improvement. We are aware that a new investigation on this topic is currently being undertaken, not restricted to PROSPERO alone and with a properly registered CSR review (Pieper et al., 2020). We hope that our findings and future research will improve reporting quality regarding changes between protocol and review.

##  Supplemental Information

10.7717/peerj.16016/supp-1 Supplemental Information 1 Search algorithm used to identify Systematic Reviews with a registered protocol on PROSPERO

10.7717/peerj.16016/supp-2 Supplemental Information 2 Magnitude of changes in relation to the quality assessed on AMSTAR-2 for non-CSRs

10.7717/peerj.16016/supp-3 Supplemental Information 3 Reporting of changes in relation to the quality assessed on AMSTAR-2 for non-CSRs

10.7717/peerj.16016/supp-4 Supplemental Information 4 Deviations from Protocol to Article.

10.7717/peerj.16016/supp-5 Supplemental Information 5 Absolute Risk Reduction Confidence Interval Calculation

## Additional Information and Declarations

### Data Deposition

The following information was supplied regarding data availability: The data is available at OSF: Siebert, Maximilian, Meisser Madera, Ivan Sola, NAUDET Florian, and Marta Roqué. 2023. “Assessing the Magnitude of Reporting Bias in a Sample of Recent Systematic Reviews - A Comparison of a Prospero and Cochrane Sample.” OSF. August 13. doi: 10.17605/OSF.IO/5YWUS.
