# Supplementary material for: Assessing the magnitude of changes from protocol to publication—a survey on Cochrane and non-Cochrane Systematic Reviews
Source: PeerJ. 2023 Oct 2;11:e16016. doi: 10.7717/peerj.16016 (PMC10552742; doi:10.7717/peerj.16016)
Supplement: Supplemental Information 1 [file peerj-11-16016-s001.docx]

(PROSPERO[tiab] OR registration[tiab]) AND (systematic[sb] OR systematic review[tiab] OR

meta analys*[tiab] OR systematic[ti] OR review[ti] OR synthes*[ti] OR searche*[tiab] OR

pool*[ti] OR database*[tiab] OR MEDLINE[tiab] OR EMBASE[tiab]) AND ("2018/01/01"[PDAT] :

"2019/01/01"[PDAT])

**Supplementary Table 1**: Search algorithm used to identify Systematic Reviews with a registered a protocol on PROSPERO
