# Supplementary figures and images for: Assessing the magnitude of changes from protocol to publication—a survey on Cochrane and non-Cochrane Systematic Reviews
Source: PeerJ. 2023 Oct 2;11:e16016. doi: 10.7717/peerj.16016 (PMC10552742; doi:10.7717/peerj.16016)

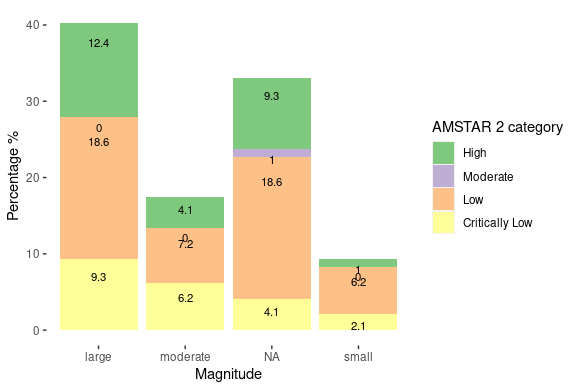

Supplement: Supplemental Information 2 [file peerj-11-16016-s002.png]

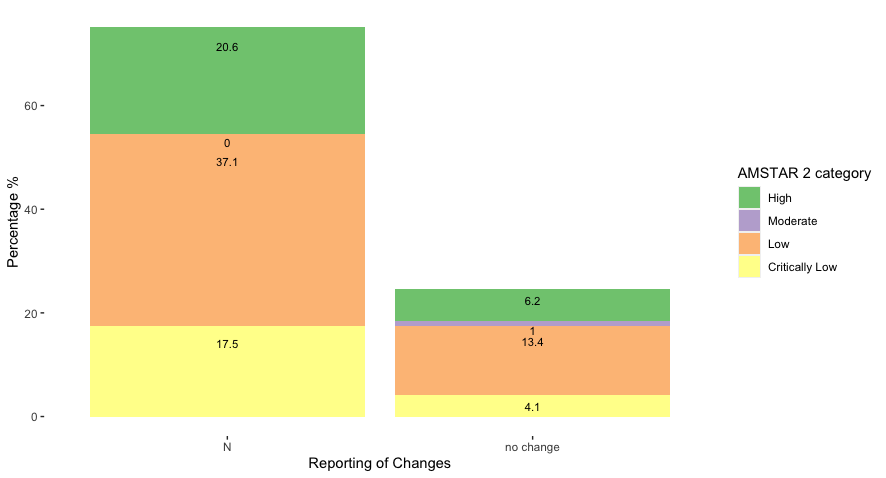

Supplement: Supplemental Information 3 [file peerj-11-16016-s003.png]
