# Supplementary material for: Assessing the magnitude of changes from protocol to publication—a survey on Cochrane and non-Cochrane Systematic Reviews
Source: PeerJ. 2023 Oct 2;11:e16016. doi: 10.7717/peerj.16016 (PMC10552742; doi:10.7717/peerj.16016)
Supplement: Supplemental Information 4 [file peerj-11-16016-s004.docx]

### Deviations from protocol to article

In the original protocol for this project, we planned to include CSRs that had reported at least one change from protocol. We realized that this would introduce a bias, and we decided to include a sample of CSRs without declared changes that would complement the first one. As some of these reviews from the second randomization were unavailable or ineligible, we substituted them for 9 CSRs without changes excluded in the first randomization.

Furthermore, during the extraction we realized it is easier to establish a binary variable for changes in the characteristics. So only if a change was present in one of the PICOS elements, we eventually described the magnitude of it.

The extraction of PICOS characteristics has been realized by two researchers independently and checked between team members. In the original protocol it was planned to have only one researcher extracting the items. We made a change here to increase quality and correctness of the research findings.

The pilot has been conducted on three studies for each review type instead of five as planned, since two of the originally planned pilot studies were not eligible. In addition, the remaining three have been part of the final analysis.

Changing of the review title and or secondary outcomes were not part of the PICOS items and thus not considered for characterizing the magnitude of change.

After peer-review, we changed the title of the project and applied hypothesis testing only to PICOS items.
