# Supplementary material for: Assessing the magnitude of changes from protocol to publication—a survey on Cochrane and non-Cochrane Systematic Reviews
Source: PeerJ. 2023 Oct 2;11:e16016. doi: 10.7717/peerj.16016 (PMC10552742; doi:10.7717/peerj.16016)
Supplement: Supplemental Information 5 [file peerj-11-16016-s005.docx]

For Absolute Risk Reduction, we used ARR = (changes in CSR)/97 – (changes in non-CSR)/97

$$ARR= \frac{CSR Changes}{97}- \frac{nonCSR}{97}$$

For the Standard Error : $\sqrt{\frac{\frac{CSR Changes}{97}\times\left( 1-\frac{CSR Changes}{97} \right)}{97}}+\frac{\frac{nonCSR}{97}\times\left( 1-\frac{nonCSR}{97} \right)}{97}$

CI confidence interval for an alpha level of 0.05 was then defined as ARR +/- 1.96 *SE
